# Endoscopic ultrasound service readiness across a provincial endoscopy network in Hubei, China: a cross-sectional institutional survey

**DOI:** 10.3389/fmed.2026.1937464

**Published:** 2026-10-14

**Authors:** Jun Zhang, Yanxia Li, Xu Huang, Honggang Yu

**Affiliations:** 1 Department of Gastroenterology, Renmin Hospital of Wuhan University, Wuhan, China; 2 Hubei Provincial Clinical Research Center for Digestive Disease Minimally Invasive Incision, Renmin Hospital of Wuhan University, Wuhan, China; 3 Engineering Research Center for Artificial Intelligence Endoscopy Interventional Treatment of Hubei Province, Renmin Hospital of Wuhan University, Wuhan, China

**Keywords:** endoscopic ultrasound, EUS-guided fine-needle aspiration, institutional survey, quality monitoring, service readiness

## Abstract

**Background:**

Endoscopic ultrasound (EUS) is essential for pancreatobiliary evaluation, subepithelial-lesion assessment, tumor staging, and tissue acquisition. We evaluated EUS service availability, institutional resources, and quality-indicator reporting across a provincial endoscopy network in Hubei, China.

**Methods:**

We conducted a cross-sectional survey of 99 digestive endoscopy institutions from January to March 2026. Institutions were classified by administrative level and four mutually exclusive capability tiers: no EUS, miniprobe-only EUS, diagnostic radial/linear EUS without EUS-FNA, and EUS-guided fine-needle aspiration (EUS-FNA). Analyses included descriptive statistics, Wilson 95% confidence intervals, and exploratory univariable associations.

**Results:**

Any EUS service was reported by 33/99 institutions (33.3%). Of these, 16 provided miniprobe-only EUS, 6 provided diagnostic radial/linear EUS without EUS-FNA, and 11 provided EUS-FNA. The diagnostic radial/linear and EUS-FNA tiers together comprised 17 institutions with radial/linear EUS capability, all of which were city-level hospitals. Median annual endoscopy volume was 1,518.5 (IQR, 309.3-4,000) in the no-EUS tier and 21,293 (IQR, 10,032-29,810.5) in the EUS-FNA tier; corresponding endoscopist counts were 4 (IQR, 2-6) and 14 (IQR, 11-19.5). The eight highest-volume institutions accounted for 81.9% of reported EUS activity. EUS completion-rate reporting was present in 30/33 EUS providers (90.9%), and EUS-FNA pathological-yield reporting in 9/11 EUS-FNA providers (81.8%).

**Conclusions:**

EUS services in the participating network were concentrated in city-level hospitals, with higher median annual endoscopy volume and endoscopist staffing in the higher capability tiers. These findings describe service provision within the surveyed institutions and identify questions for future evaluation of referral, training, and quality monitoring.

## Introduction

Endoscopic ultrasound (EUS) has become a core modality in gastrointestinal diagnosis and advanced endoscopy. By combining endoscopic visualization with high-resolution ultrasonography, EUS supports evaluation of the gastrointestinal wall, adjacent structures, lymph nodes, and the pancreatobiliary system. It is central to the assessment of subepithelial lesions, pancreatic cysts, suspected pancreatobiliary malignancy, tumor staging, and selected therapeutic procedures (1–3). The expansion of EUS-guided tissue acquisition has further increased its importance in contemporary digestive disease care (3–5).

EUS service delivery is more resource-intensive than routine diagnostic endoscopy. Reliable practice requires specialized equipment, trained endoscopists, adequate case volume, sedation or anesthesia support, specimen-handling pathways, pathology coordination, and continuous quality monitoring (6–9). These requirements are especially important for EUS-guided fine-needle aspiration or biopsy, for which diagnostic yield depends on case selection, sampling technique, needle choice, specimen processing, cytopathology support, and feedback to the endoscopist (10–13).

EUS services encompass different levels of clinical function, infrastructure demand, and risk management. Miniprobe-only EUS supports selected superficial or subepithelial-lesion assessment. Radial and linear echoendoscopes permit more extensive evaluation of adjacent organs, while EUS-guided tissue acquisition requires linear-array guidance and appropriate specimen handling. Quality indicators need to reflect the actual scope of service, including completion, diagnostic adequacy, tissue yield, adverse-event tracking, and documentation (9, 14, 15). Distinguishing these functions can help characterize the services that institutions report providing and the support needed for each level of practice.

This distinction is particularly relevant in provincial health systems that include city-level hospitals, county-level hospitals, and township or community institutions. Hubei, a province in central China, had 58.11 million permanent residents and 39,517 health-care institutions at the end of 2025, including 1,276 hospitals and 37,698 primary health-care institutions. National studies describe expansion of health-care coverage alongside continuing differences in access and resource distribution (16, 17). Within this context, the objective of the present study was to describe EUS service availability and capability tiers among institutions participating in the Hubei digestive endoscopy quality-control survey, examine their institutional resource patterns, and assess recording of service-specific quality indicators.

## Methods

### Study design and data source

We conducted a cross-sectional institutional analysis using data from the Hubei Province digestive endoscopy quality-control survey administered from January to March 2026. The analytic dataset comprised records from 99 institutions, all of which were included in the analysis. The questionnaire collected information on endoscopy infrastructure, staffing, procedure volumes, EUS equipment, EUS and EUS-FNA service activity, hospital characteristics, and selected quality-reporting indicators. It did not include patient identifiers, lesion-level findings, waiting times, EUS-related adverse events, subsequent management, or clinical outcomes. Procedure volumes were analyzed as annual volumes reported by each institution; however, the source dataset did not identify the calendar year to which these volumes referred. Reporting was guided by the Consensus-Based Checklist for Reporting of Survey Studies (CROSS) (18).

### Institutional classification

Institutions were classified by administrative level as city-level hospitals, county-level hospitals, or township/community institutions. A county-facing variable combined county-level and township/community institutions. Administrative level was analyzed separately from hospital accreditation grade. Hospital type was classified as general hospital, traditional Chinese medicine hospital, or township/community institution. Prefecture-level identifiers were not used for between-prefecture comparisons.

### Definitions of EUS service and capability

The primary service outcome was availability of any EUS service, defined as a positive sum of reported annual miniprobe, linear-array, and radial EUS procedure volumes. EUS equipment availability was defined as a positive entry for at least one ultrasound miniprobe, radial echoendoscope, or linear echoendoscope. Radial/linear equipment availability was derived only from the corresponding equipment fields. EUS-FNA availability was defined as a positive annual EUS-FNA procedure volume. EUS-FNA counts were analyzed separately and were not added to total EUS volume, to avoid potentially counting the same procedure twice.

Each institution was assigned to one of four mutually exclusive tiers according to its highest reported EUS capability. Institutions reporting an annual EUS-FNA procedure volume greater than zero were classified in the EUS-FNA tier. Among the remaining institutions, those with radial or linear echoendoscope equipment were classified in the diagnostic radial/linear EUS tier. Institutions with miniprobe equipment or reported EUS activity, but without radial/linear equipment or EUS-FNA activity, were classified in the miniprobe-only EUS tier. Institutions reporting neither EUS equipment nor EUS activity were classified in the no-EUS tier. Because EUS-FNA requires linear-array EUS guidance, institutions in the EUS-FNA tier were considered to have radial/linear-or-higher capability.

### Data cleaning

All institutional records were reviewed before analysis. Of the 99 institutions, 29 contained at least one text-formatted response in the equipment, institutional resource, staffing, or EUS procedure-volume fields. Numerical values accompanied by units were converted to numeric format. Ranges were represented by their midpoints, approximate values were recorded as the stated numerical value, and clear typographical substitutions were standardized. Entries explicitly indicating the absence of equipment, staff, or procedural activity were coded as zero. No values were imputed from other institutions; entries that could not be interpreted numerically were retained as missing for the corresponding variable. Anesthesia support was excluded from quantitative analyses because it was not collected as a standardized numerical staffing count.

### Quality-reporting indicators

Quality reporting was evaluated using two institutional indicators: EUS completion-rate reporting and EUS-FNA pathological-yield reporting. The EUS-FNA pathological-yield item recorded the proportion of patients with positive cytological or histopathological findings among those undergoing EUS-FNA for clinically suspected pancreatic malignancy. Each indicator was evaluated among institutions reporting activity for the corresponding procedure. An indicator was classified as reported when the institution provided a positive numerical rate in the relevant survey field. The results were expressed as the proportion of eligible institutions reporting each indicator.

### Statistical analysis

Categorical variables were summarized as counts and percentages, with Wilson 95% confidence intervals (CIs) calculated for proportions (19, 20). Wilson intervals were used to describe the precision of individual proportions and were not interpreted as hypothesis tests. Continuous variables were summarized as medians and interquartile ranges (IQRs; 25th-75th percentiles), with quantiles estimated by linear interpolation. Institutional resources were compared descriptively across the four EUS capability tiers. Exploratory univariable associations were examined for three outcomes: any EUS service, radial/linear equipment availability, and EUS-FNA service. Associations were expressed as odds ratios (ORs) with 95% CIs, and two-sided Fisher exact tests were used to calculate *P* values (21, 22). For estimation of ORs and log-Wald 95% CIs, 0.5 was added to each cell of the corresponding 2 × 2 table; Fisher exact tests were based on the observed cell counts. Analyses were limited to univariable estimates, and *P* values were not adjusted for multiple comparisons. All analyses were conducted using R version 4.1.2 (R Foundation for Statistical Computing, Vienna, Austria).

### Ethics

This study analyzed anonymized institution-level data collected through a provincial quality-control survey and did not include patient-level or personally identifiable information. Because the analysis was limited to institution-level quality-control data and did not involve human-participant data, ethics committee approval and individual informed consent were not required.

## Results

### Study population and overall EUS capacity

The analysis included 99 digestive endoscopy institutions across the provincial network, comprising 40 city-level hospitals, 40 county-level hospitals, and 19 township/community institutions. By hospital type, 60 institutions were general hospitals, 20 were traditional Chinese medicine hospitals, and 19 were township/community institutions.

Any EUS equipment and any annual EUS service volume were each reported by 33/99 institutions (33.3%; 95% CI, 24.8-43.1). Radial or linear echoendoscopes were available in 17/99 institutions (17.2%; 95% CI, 11.0-25.8), and EUS-FNA service was reported by 11/99 (11.1%; 95% CI, 6.3-18.8). Figure 1 summarizes three network-level EUS capacity indicators across the surveyed institutions.

**Figure 1:**
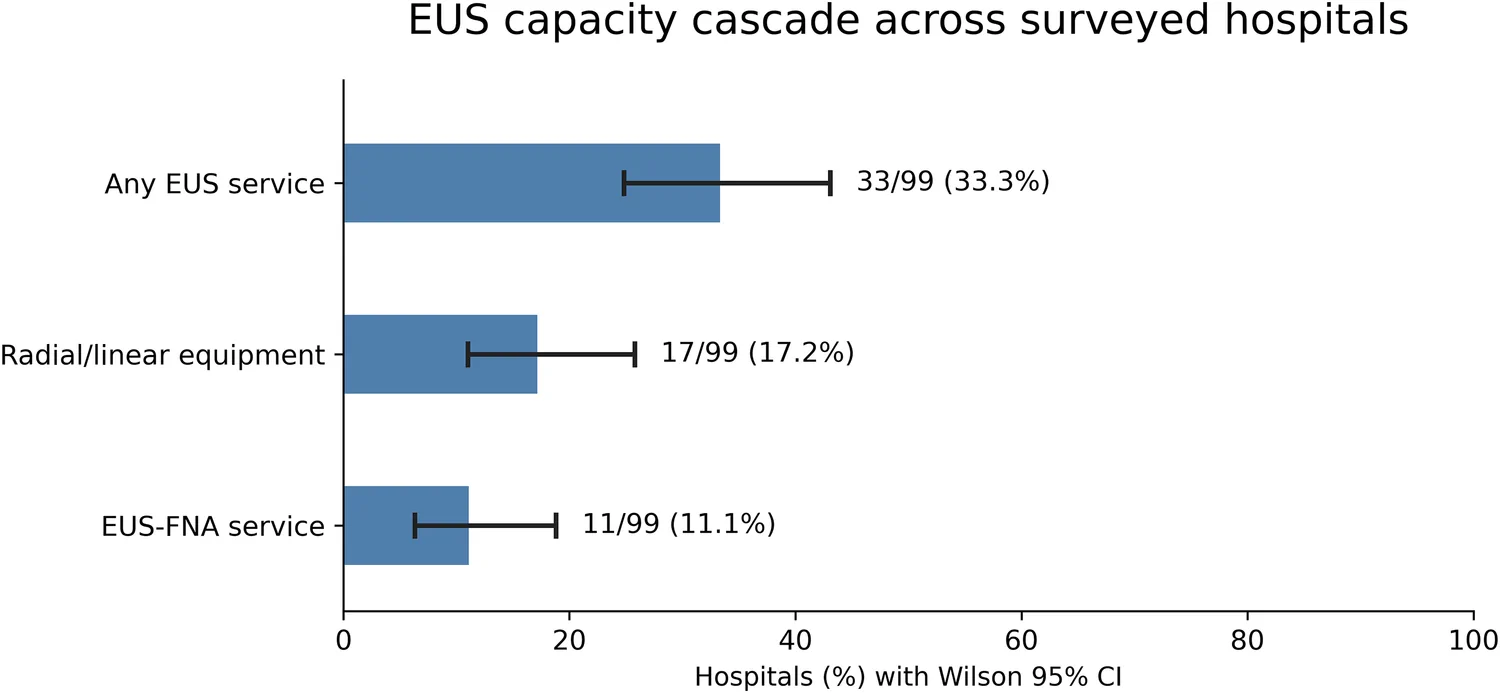
EUS capacity cascade across 99 surveyed digestive endoscopy institutions. Bars show the proportion of institutions with any EUS service, radial/linear equipment, and EUS-FNA service; error bars indicate Wilson 95% confidence intervals. EUS, endoscopic ultrasound; EUS-FNA, endoscopic ultrasound-guided fine-needle aspiration.

### Administrative gradient and capability tiers

EUS service availability varied across administrative levels. Among the 40 city-level hospitals, 26 provided EUS services (65.0%; 95% CI, 49.5%-77.9%), 17 had radial/linear equipment (42.5%; 95% CI, 28.5%-57.8%), and 11 provided EUS-FNA services (27.5%; 95% CI, 16.1%-42.8%). Among the 40 county-level hospitals, 7 provided EUS services (17.5%; 95% CI, 8.7%-31.9%), whereas none had radial/linear equipment or provided EUS-FNA services. None of the 19 township or community institutions provided EUS services (Table 1 and Figure 2).

**Table 1:** EUS service availability by administrative level.

Group	Institutions, n	Any EUS service	Radial/linear equipment	EUS-FNA service	Endoscopy volume, n	EUS volume, n	Endoscopists, n	Center area, m^2^
City-level	40	65.0 (49.5-77.9)	42.5 (28.5-57.8)	27.5 (16.1-42.8)	7,626 (5,684.8-18,016.5)	43 (0-143.3)	9 (6.8-12.3)	500 (254.9-804.5)
County-level	40	17.5 (8.7-31.9)	0.0 (0.0-8.8)	0.0 (0.0-8.8)	3,281 (1,400-5,238.3)	0 (0-0)	4 (3-6)	200 (107.5-305)
Township/community	19	0.0 (0.0-16.8)	0.0 (0.0-16.8)	0.0 (0.0-16.8)	200 (138.5-250)	0 (0-0)	2 (1-2.5)	40 (30-70)

Categorical data are % (Wilson 95% CI). Resource variables are median (IQR).

EUS, endoscopic ultrasound; EUS-FNA, endoscopic ultrasound-guided fine-needle aspiration; IQR, interquartile range.

**Figure 2:**
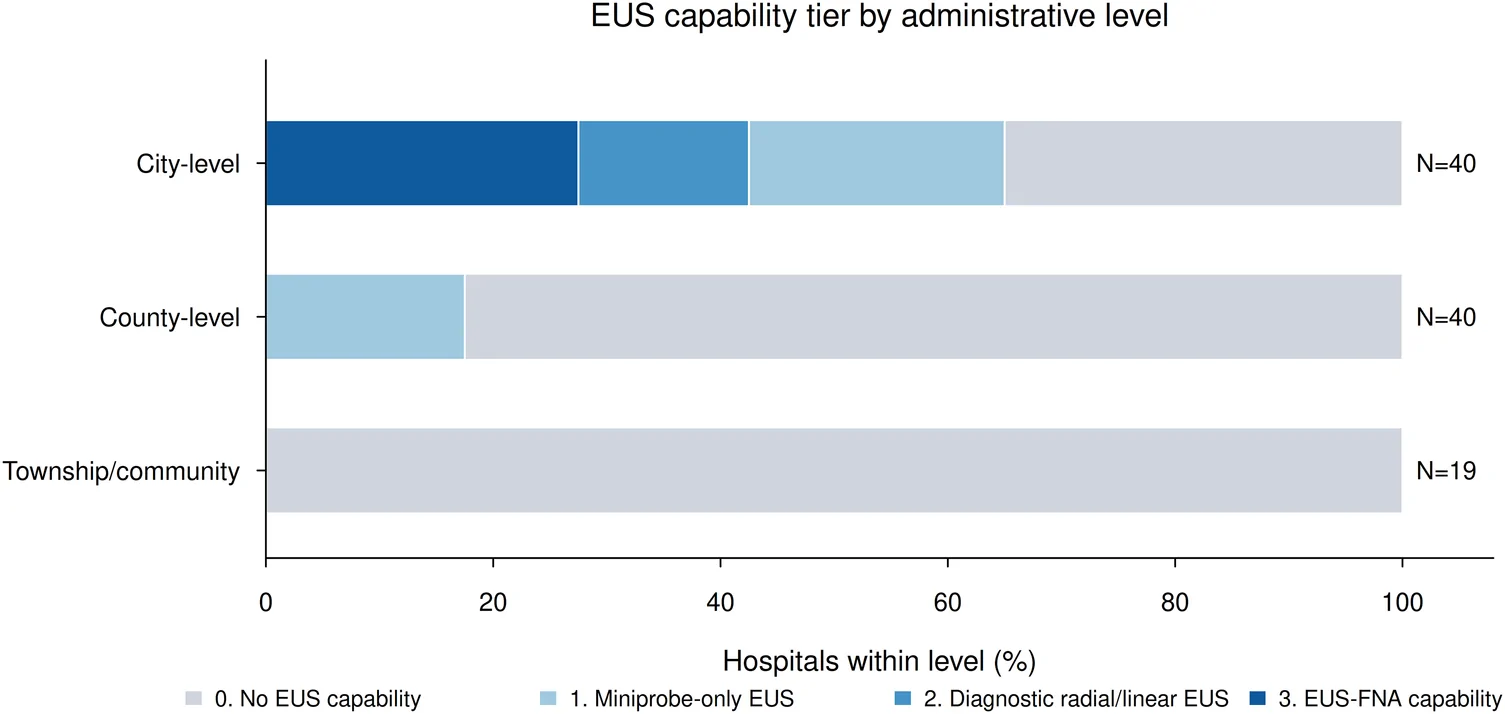
Distribution of mutually exclusive EUS capability tiers by administrative level. Bar segments represent the proportion of institutions within each administrative stratum assigned to no EUS, miniprobe-only EUS, diagnostic radial/linear EUS without EUS-FNA, or EUS-FNA. EUS, endoscopic ultrasound; EUS-FNA, endoscopic ultrasound-guided fine-needle aspiration.

Across the 99 institutions, the four mutually exclusive capability tiers comprised 66 institutions with no EUS, 16 with miniprobe-only EUS, 6 with diagnostic radial/linear EUS without EUS-FNA, and 11 with EUS-FNA. The diagnostic radial/linear and EUS-FNA tiers together comprised 17 institutions with radial/linear EUS capability, all of which were city-level hospitals. Of the 26 city-level EUS providers, 9 were classified in the miniprobe-only tier, 6 in the diagnostic radial/linear tier, and 11 in the EUS-FNA tier. All 7 county-level EUS providers were classified in the miniprobe-only tier, and all township or community institutions were classified in the no-EUS tier (Figure 2). EUS services were reported by 30 of 60 general hospitals (50.0%) and 3 of 20 traditional Chinese medicine hospitals (15.0%) (Supplementary Table S1).

### Resource gradients and concentration of EUS activity

Median annual endoscopy volume was 1,518.5 (IQR, 309.3-4,000), 6,536.5 (IQR, 5,146-7,492), 16,000 (IQR, 12,222.5-18,049.5), and 21,293 (IQR, 10,032-29,810.5) across the no-EUS, miniprobe-only, diagnostic radial/linear, and EUS-FNA tiers, respectively. Median center area was 120 m^2^ (IQR, 50.5-275), 340 m^2^ (IQR, 200-500), 550 m^2^ (IQR, 500-1,275), and 900 m^2^ (IQR, 403-1,350). Total endoscopist staffing and postgraduate physician counts also increased across these tiers (Table 2). Senior physician counts were higher in the two upper tiers but did not increase monotonically. Anesthesia staffing was not included in these comparisons.

**Table 2:** Institutional resources across mutually exclusive EUS capability tiers.

Capability tier	Institutions, n	Annual endoscopy volume	Center area, m^2^	Endoscopists, n	Senior physicians, n	Postgraduate physicians, n
No EUS	66	1,518.5 (309.3-4,000)	120 (50.5-275)	4 (2-6)	1 (0-2)	0 (0-0)
Miniprobe-only EUS	16	6,536.5 (5,146-7,492)	340 (200-500)	8 (6-9)	2 (1.8-4)	1 (0-3)
Diagnostic radial/linear EUS without EUS-FNA	6	16,000 (12,222.5-18,049.5)	550 (500-1,275)	12.5 (11.3-13.8)	6 (6-6.8)	7.5 (4.5-9.8)
EUS-FNA	11	21,293 (10,032-29,810.5)	900 (403-1,350)	14 (11-19.5)	5 (4-7.5)	9 (5.5-14.5)

Values are median (IQR) unless otherwise indicated. Capability tiers are mutually exclusive. The EUS-FNA tier was assigned by positive annual EUS-FNA procedure volume and took precedence over equipment-based tier assignment. EUS, endoscopic ultrasound; EUS-FNA, endoscopic ultrasound-guided fine-needle aspiration; IQR, interquartile range.

EUS activity was unevenly distributed across institutions. The eight highest-volume institutions accounted for 81.9% of the reported annual EUS procedure volume (Figure 3). Exploratory univariable analyses identified associations between EUS availability and city-level status, general-hospital status, higher annual endoscopy volume, larger center area, greater endoscopist staffing, and postgraduate physician availability. For example, any EUS service was reported by 26/40 city-level and 7/59 county-level or township/community institutions (corrected OR, 12.79; 95% CI, 4.72-34.69; Fisher *P* < 0.001). Full event counts, ORs, CIs, and *P* values are provided in Supplementary Table S2.

**Figure 3:**
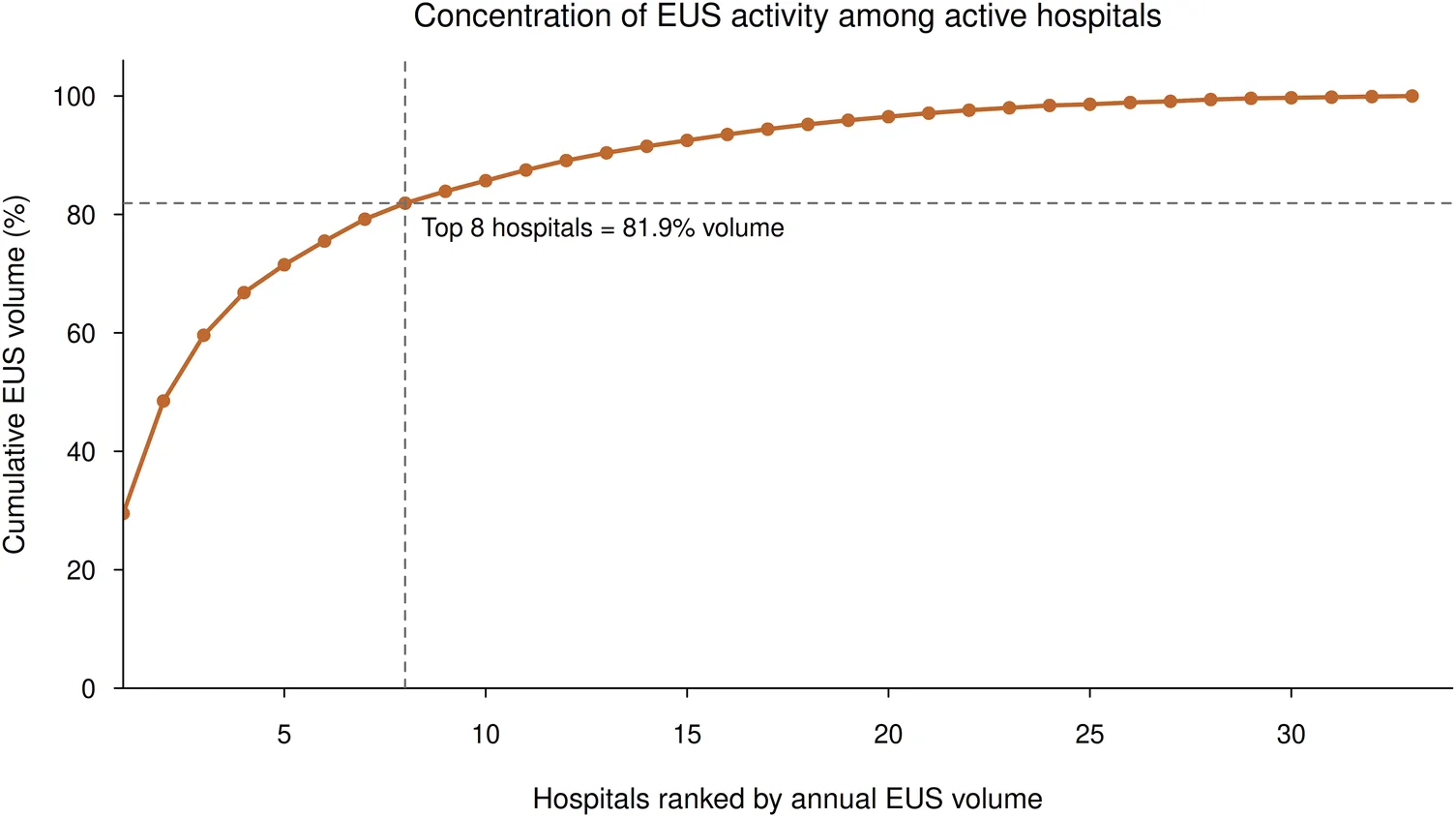
Concentration of annual EUS procedure volume among active institutions. The cumulative proportion of annual EUS volume is shown after institutions are ranked from highest to lowest reported volume. The top eight institutions accounted for 81.9% of reported annual EUS volume. EUS, endoscopic ultrasound.

### Provider-level quality reporting

Among the 33 institutions providing EUS services, 30 reported an EUS completion rate (90.9%; 95% CI, 76.4%-96.9%). Among the 11 institutions providing EUS-FNA, 9 reported the pathological yield for EUS-FNA performed in patients with clinically suspected pancreatic malignancy (81.8%; 95% CI, 52.3%-94.9%). These indicators were summarized using institutions providing the corresponding service as the denominator.

## Discussion

This survey describes EUS service provision across 99 digestive endoscopy institutions in Hubei Province. EUS was reported by 33 institutions, and 11 provided EUS-FNA. All 17 institutions with radial/linear EUS capability were city-level hospitals; the seven county-level EUS providers were confined to the miniprobe-only tier, and no township or community institution reported EUS activity. Higher capability tiers also had greater endoscopy volume, larger center area, and more endoscopists. In addition, eight institutions accounted for 81.9% of reported annual EUS volume. Together, these findings show a service structure in which both technical capability and procedural activity were concentrated in a limited number of higher-level institutions.

The four-tier classification helps distinguish services that would otherwise be combined under a single EUS availability measure. Miniprobe EUS is mainly used for superficial and intramural lesions and can be incorporated into conventional endoscopy workflows. Radial and linear echoendoscopes extend examination to deeper structures and the pancreatobiliary system, while EUS-FNA adds tissue acquisition and requires experience in lesion targeting, needle selection, specimen acquisition, processing, and pathological feedback (1, 2, 5, 9). In this survey, 16 of the 33 EUS providers were limited to miniprobe EUS, 6 provided diagnostic radial/linear EUS without EUS-FNA, and 11 provided EUS-FNA. The tier distribution therefore adds clinically relevant information beyond a binary measure of EUS availability.

The concentration of conventional and interventional EUS in city-level hospitals is consistent with national data from China. National surveys documented substantial growth in EUS utilization between 2012 and 2019, while EUS activity remained concentrated in higher-level hospitals and varied markedly across provincial regions (23). The present study provides a provincial view of this pattern by separating miniprobe-only activity, diagnostic radial/linear EUS, and EUS-guided tissue acquisition. This distinction is useful when service availability is interpreted in relation to the procedures an institution can actually provide.

The administrative gradient has practical implications for patient pathways. County-level institutions represented an entry point for basic EUS services, while conventional radial/linear EUS and EUS-FNA were provided by city-level centers. Patients requiring pancreatobiliary evaluation or tissue acquisition may therefore move between institutions with different levels of capability. Effective coordination in this setting depends on appropriate identification of indications, transfer of prior imaging and clinical information, timely scheduling, pathology processing, and communication of results to the referring institution. Recording referral origin, destination, waiting time, repeat examinations, and completion of follow-up would allow future studies to evaluate how the current distribution of services affects continuity and timeliness of care.

A coordinated referral network offers a plausible framework for linking institutions with different capabilities. County-level providers could contribute to initial assessment, identification of patients requiring advanced EUS, preparation of referral information, and follow-up after the procedure. City-level centers could provide conventional diagnostic EUS, tissue acquisition, pathology coordination, and training support. Experience from other technically demanding areas of care suggests that procedural volume, specialist expertise, and multidisciplinary support often develop together (24–26). Evaluation of an EUS referral network should include access, waiting time, travel burden, diagnostic completion, repeat testing, and clinical outcomes. These measures would permit comparison of alternative service arrangements within the provincial networkSeveral measured institutional resources were higher across the ordered capability tiers. Median annual endoscopy volume rose from 1,518.5 procedures in the no-EUS tier to 21,293 procedures in the EUS-FNA tier, and the median number of endoscopists increased from 4 to 14. Center area and postgraduate physician staffing showed similar gradients. In this cross-sectional survey, higher EUS capability was observed in institutions with larger clinical workloads and broader staffing bases. These associations do not establish the direction of causation: larger endoscopy programs may provide more opportunities for supervised training and service support, while established EUS services may also attract referrals and contribute to subsequent growth in workload and staffing. Longitudinal data would help clarify how these institutional characteristics evolve during service development.

The concentration of 81.9% of annual EUS activity in eight institutions adds an important dimension to the availability data. Institutional availability indicates where a service is offered, whereas procedure volume reflects how frequently that service is used. A network may contain several EUS providers while most examinations remain concentrated in a small group of centers. This pattern has implications for training exposure, maintenance of procedural skills, equipment use, and workload distribution. Future surveys could record the number of independent EUS operators, annual volume per operator, types of procedures performed, and the proportion of examinations referred from other institutions. These data would provide a clearer basis for assessing workforce requirements and training capacity.

Training and quality assessment should reflect the procedures performed at each capability tier. Miniprobe EUS requires competence in lesion localization and wall-layer assessment; conventional radial or linear EUS requires systematic examination of pancreatobiliary and upper abdominal anatomy; and EUS-FNA adds needle control, sampling strategy, specimen acquisition, and adverse-event management. Current guidance emphasizes structured training and objective assessment of competence in advanced endoscopy (14, 15). The present survey did not collect operator-level training histories, supervised procedure counts, or independent-practice status, and these variables would strengthen future workforce assessments.

Most institutions providing EUS services recorded the corresponding quality indicators. EUS completion-rate data were reported by 90.9% of EUS providers, and pathological-yield data were reported by 81.8% of EUS-FNA providers. This level of participation provides a foundation for routine quality monitoring across the network. Completion rate reflects the consistency of the examination process, while pathological yield captures an important component of tissue-acquisition performance. Shared definitions, explicit patient-level numerators and denominators, and regular review of submitted data would improve comparability across institutions and survey cycles. Quality monitoring could be expanded to include specimen adequacy, diagnostic accuracy, adverse events, pathology turnaround time, and communication of results, in accordance with current EUS quality guidance (9).

Repeated provincial surveys could use the same tier structure to track changes in service scope, annual procedure volume, staffing, and operator expertise. Separate fields for miniprobe, radial, linear, tissue-acquisition, and therapeutic EUS would allow each component of service capability to be followed over time. Recording equipment ownership, functional status, shared access, and actual use would also reduce ambiguity when equipment data are compared with reported procedural activity.

This study has several strengths. Equipment, procedural activity, staffing, endoscopy workload, and institutional characteristics were assessed within the same dataset, and the survey included city-level, county-level, and township or community institutions. The mutually exclusive tiers provided a consistent framework for describing basic EUS activity, conventional diagnostic capability, and tissue-acquisition services. The analysis also used service-specific denominators for quality reporting and quantified the concentration of procedural activity among the highest-volume institutions.

Several limitations should be considered. The cross-sectional design captured institutional services during a single survey period and did not include longitudinal changes in capability. Patient-level information on indications, referral pathways, waiting times, diagnostic performance, adverse events, subsequent management, and clinical outcomes was unavailable. The survey records did not provide the eligible sampling frame or invitation denominator, which prevented calculation of the response rate and population coverage. Procedure volumes and institutional resources were self-reported, and the reference year for annual procedure volumes was not specified in the analytic dataset. The EUS-FNA tier contained 11 institutions, resulting in limited precision for some exploratory estimates. The univariable analyses used sample-derived thresholds and did not account for correlations among institutional volume, staffing, center size, and hospital type. These features define the scope of the findings and identify priorities for subsequent data collection.

In conclusion, EUS services within the surveyed endoscopy network were concentrated in city-level hospitals, with miniprobe-only activity reported at county-level institutions. Higher capability tiers generally coincided with larger centers, greater endoscopy volume, and more endoscopists. The study identifies differences in reported service scope and resources that can guide subsequent assessment of training needs, referral processes, and quality monitoring. Prospective studies incorporating patient access, procedural performance, and clinical outcomes are needed to evaluate service organization and the effects of any planned expansion.

## Data Availability

The original contributions presented in the study are included in the article/Supplementary Material, further inquiries can be directed to the corresponding author.

## References

[1] Jacobson BC Bhatt A Greer KB Lee LS Park WG Sauer BG. ACG clinical guideline: diagnosis and management of gastrointestinal subepithelial lesions. Am J Gastroenterol. (2023) 118(1):46–58. 10.14309/ajg.0000000000002100 36602835

[2] Deprez PH Moons LMG O'Toole D Gincul R Seicean A Pimentel-Nunes P. Endoscopic management of subepithelial lesions including neuroendocrine neoplasms: european society of gastrointestinal endoscopy (ESGE) guideline. Endoscopy. (2022) 54(4):412–29. 10.1055/a-1751-5742 35180797

[3] van Der Merwe SW Van Wanrooij RLJ Bronswijk M Everett S Lakhtakia S Rimbas M. Therapeutic endoscopic ultrasound: european society of gastrointestinal endoscopy (ESGE) guideline. Endoscopy. (2022) 54(2):185–205. 10.1055/a-1717-1391 34937098

[4] Polkowski M Jenssen C Kaye P Carrara S Deprez P Gines A. Technical aspects of endoscopic ultrasound (EUS)-guided sampling in gastroenterology: european society of gastrointestinal endoscopy (ESGE) technical guideline - march 2017. Endoscopy. (2017) 49(10):989–1006. 10.1055/s-0043-119219 28898917

[5] Eloubeidi MA Decker GA Chandrasekhara V Chathadi KV Early DS Evans JA. The role of endoscopy in the evaluation and management of patients with solid pancreatic neoplasia. Gastrointest Endosc. (2016) 83(1):17–28. 10.1016/j.gie.2015.09.009 26706297

[6] Hewitt MJ McPhail MJ Possamai L Dhar A Vlavianos P Monahan KJ. EUS-guided FNA for diagnosis of solid pancreatic neoplasms: a meta-analysis. Gastrointest Endosc. (2012) 75(2):319–31. 10.1016/j.gie.2011.08.049 22248600

[7] Banafea O Mghanga FP Zhao J Zhao R Zhu L. Endoscopic ultrasonography with fine-needle aspiration for histological diagnosis of solid pancreatic masses: a meta-analysis of diagnostic accuracy studies. BMC Gastroenterol. (2016) 16(1):108. 10.1186/s12876-016-0519-z 27580856 PMC5007683

[8] Wang K-X Ben Q-W Jin Z-D Du Y-Q Zou D-W Liao Z. Assessment of morbidity and mortality associated with EUS-guided FNA: a systematic review. Gastrointest Endosc. (2011) 73(2):283–90. 10.1016/j.gie.2010.10.045 21295642

[9] Mishra G Lennon AM Pausawasdi N Shami VM Sharaiha RZ Elmunzer BJ. Quality indicators for EUS. Gastrointest Endosc. (2025) 101(5):928–49.e1. 10.1016/j.gie.2025.02.025 40266165

[10] Bang JY Hebert-Magee S Hasan MK Navaneethan U Hawes R Varadarajulu S. Endoscopic ultrasonography-guided biopsy using a franseen needle design: initial assessment. Dig Endosc. (2017) 29(3):338–46. 10.1111/den.12769 27878861

[11] Verloop CA Goos JAC Bruno MJ Quispel R Van Driel L Hol L. Diagnostic yield of endoscopic and EUS-guided biopsy techniques in subepithelial lesions of the upper GI tract: a systematic review. Gastrointest Endosc. (2024) 99(6):895–911.e13. 10.1016/j.gie.2024.02.003 38360118

[12] Chen T-Y Cao J-W Jin C Ji Y Zhong L Wang L-M. Comparison of specimen quality among the standard suction, slow-pull, and wet suction techniques for EUS-FNA: a multicenter, prospective, randomized controlled trial. Endosc Ultrasound. (2022) 11(5):393–400. 10.4103/EUS-D-21-00163 36255027 PMC9688131

[13] Facciorusso A Del Prete V Buccino VR Purohit P Setia P Muscatiello N. Diagnostic yield of franseen and fork-tip biopsy needles for endoscopic ultrasound-guided tissue acquisition: a meta-analysis. Endosc Int Open. (2019) 7(10):E1221–e30. 10.1055/a-0982-2997 31579703 PMC6773615

[14] Wani S Cote GA Keswani RN Yadlapati RH Hall M O’Hara J. Development of American society for gastrointestinal endoscopy standards for training in advanced endoscopy within dedicated advanced endoscopy fellowship programs. Gastrointest Endosc. (2025) 101(1):12–24. 10.1016/j.gie.2024.03.025 38935016 PMC13277225

[15] Ekkelenkamp VE Koch AD De Man RA Kuipers EJ. Training and competence assessment in GI endoscopy: a systematic review. Gut. (2016) 65(4):607–15. 10.1136/gutjnl-2014-307173 25636697

[16] Yip W Fu H Chen AT Zhai T Jian W Xu R. 10 Years of health-care reform in China: progress and gaps in universal health coverage. Lancet. (2019) 394(10204):1192–204. 10.1016/S0140-6736(19)32136-1 31571602

[17] Meng Q Xu L Zhang Y Qian J Cai M Xin Y. Trends in access to health services and financial protection in China between 2003 and 2011: a cross-sectional study. Lancet. (2012) 379(9818):805–14. 10.1016/S0140-6736(12)60278-5 22386034

[18] Sharma A Minh Duc NT Luu Lam Thang T Nam NH Ng SJ Abbas KS. A consensus-based checklist for reporting of survey studies (CROSS). J Gen Intern Med. (2021) 36(10):3179–87. 10.1007/s11606-021-06737-1 33886027 PMC8481359

[19] Wilson EB. Probable inference, the law of succession, and statistical inference. J Am Stat Assoc. (1927) 22(158):209–12. 10.1080/01621459.1927.10502953

[20] Agresti A Coull BA. Approximate is better than “exact” for interval estimation of binomial proportions. Am Stat. (1998) 52(2):119–26. 10.1080/00031305.1998.10480550

[21] Haldane JB. The estimation and significance of the logarithm of a ratio of frequencies. Ann Hum Genet. (1956) 20(4):309–11. 10.1111/j.1469-1809.1955.tb01285.x 13314400

[22] Anscombe FJ. On estimating binomial response relations. Biometrika. (1956) 43(3-4):461–4. 10.1093/biomet/43.3-4.461

[23] Xin L Gao Y Wang T-J Meng Q-Q Jin Z-D Fu Z-J. EUS development in China: results from national surveys in 2013 and 2020. Endosc Ultrasound. (2023) 12(1):90–5. 10.4103/EUS-D-22-00003 36861507 PMC10134942

[24] Birkmeyer JD Siewers AE Finlayson EVA Stukel TA Lucas FL Batista I. Hospital volume and surgical mortality in the United States. N Engl J Med. (2002) 346(15):1128–37. 10.1056/NEJMsa012337 11948273

[25] Gooiker GA Van Gijn W Wouters MW Post PN Van De Velde CJ Tollenaar RA. Systematic review and meta-analysis of the volume-outcome relationship in pancreatic surgery. Br J Surg. (2011) 98(4):485–94. 10.1002/bjs.7413 21500187

[26] Hata T Motoi F Ishida M Naitoh T Katayose Y Egawa S. Effect of hospital volume on surgical outcomes after pancreaticoduodenectomy: a systematic review and meta-analysis. Ann Surg. (2016) 263(4):664–72. 10.1097/SLA.0000000000001437 26636243

