# Supplementary material for: Endoscopic ultrasound service readiness across a provincial endoscopy network in Hubei, China: a cross-sectional institutional survey
Source: Front Med (Lausanne). 2026 Oct 14;13:1937464. doi: 10.3389/fmed.2026.1937464 (PMC13713730; doi:10.3389/fmed.2026.1937464)
Supplement: Supplementary file 1 [file Table1.docx]

**Supplementary Material**

**Table S1. EUS service availability and institutional resources by hospital type**

| **Group** | **Hospitals, n** | **Any EUS service** | **Radial/linear equipment** | **EUS-FNA service** | **Endoscopy volume, n** | **EUS volume, n** | **Endoscopists, n** | **Center area, m^2^** |
| --- | --- | --- | --- | --- | --- | --- | --- | --- |
| General hospital | 60 | 50.0  (37.7-62.3) | 26.7  (17.1-39.0) | 16.7  (9.3-28.0) | 6,537  (3,069.8-11,038.8) | 2.5  (0-73.5) | 6  (4-11.3) | 330  (150-596.3) |
| Traditional Chinese medicine hospital | 20 | 15.0  (5.2-36.0) | 5.0  (0.9-23.6) | 5.0  (0.9-23.6) | 2,039  (1,157.5-5,216) | 0  (0-0) | 5  (4-7) | 269.1  (150-340) |
| Township/community institution | 19 | 0.0  (0.0-16.8) | 0.0  (0.0-16.8) | 0.0  (0.0-16.8) | 200  (138.5-250) | 0  (0-0) | 2  (1-2.5) | 40  (30-70) |

Values for institutional resources are median (IQR). EUS, endoscopic ultrasound; EUS-FNA, endoscopic ultrasound-guided fine-needle aspiration; CI, confidence interval; IQR, interquartile range.

**Table S2. Exploratory univariable institutional correlates of EUS capacity indicators**

| **Outcome** | **Predictor** | **Events with predictor** | **Events without predictor** | **OR (95% CI)** | ***P* value** |
| --- | --- | --- | --- | --- | --- |
| Any EUS service | City-level hospital | 26/40 | 7/59 | 12.79 (4.72-34.69) | <.001 |
| Any EUS service | County-facing institution | 7/59 | 26/40 | 0.08 (0.03-0.21) | <.001 |
| Any EUS service | General hospital | 30/60 | 3/39 | 10.43 (3.12-34.84) | <.001 |
| Any EUS service | Traditional Chinese medicine hospital | 3/20 | 30/79 | 0.32 (0.09-1.11) | 0.065 |
| Any EUS service | Annual endoscopy volume≥4,000 | 32/50 | 1/49 | 56.80 (10.15-317.92) | <.001 |
| Any EUS service | Center area ≥200 m^2^ | 30/56 | 3/43 | 13.32 (3.97-44.65) | <.001 |
| Any EUS service | Endoscopists≥5 | 30/53 | 3/46 | 16.13 (4.79-54.35) | <.001 |
| Any EUS service | Any postgraduate physician | 27/38 | 6/61 | 20.42 (7.04-59.22) | <.001 |
| Any EUS service | Any senior physician | 32/75 | 1/24 | 11.70 (2.11-64.85) | <.001 |
| Radial/linear equipment | City-level hospital | 17/40 | 0/59 | 88.62 (5.12-1534.21) | <.001 |
| Radial/linear equipment | County-facing institution | 0/59 | 17/40 | 0.01 (0.00-0.20) | <.001 |
| Radial/linear equipment | General hospital | 16/60 | 1/39 | 9.52 (1.69-53.48) | 0.002 |
| Radial/linear equipment | Traditional Chinese medicine hospital | 1/20 | 16/79 | 0.30 (0.05-1.70) | 0.182 |
| Radial/linear equipment | Annual endoscopy volume ≥4,000 | 17/50 | 0/49 | 51.72 (3.01-889.76) | <.001 |
| Radial/linear equipment | Center area≥200 m^2^ | 17/56 | 0/43 | 38.54 (2.24-662.35) | <.001 |
| Radial/linear equipment | Endoscopists≥5 | 17/53 | 0/46 | 44.59 (2.59-766.54) | <.001 |
| Radial/linear equipment | Any postgraduate physician | 17/38 | 0/61 | 100.12 (5.77-1737.28) | <.001 |
| Radial/linear equipment | Any senior physician | 17/75 | 0/24 | 14.66 (0.85-253.53) | 0.010 |
| EUS-FNA service | City-level hospital | 11/40 | 0/59 | 46.39 (2.64-814.64) | <.001 |
| EUS-FNA service | County-facing institution | 0/59 | 11/40 | 0.02 (0.00-0.38) | <.001 |
| EUS-FNA service | General hospital | 10/60 | 1/39 | 5.34 (0.92-31.06) | 0.046 |
| EUS-FNA service | Traditional Chinese medicine hospital | 1/20 | 10/79 | 0.51 (0.09-3.03) | 0.454 |
| EUS-FNA service | Annual endoscopy volume ≥4,000 | 11/50 | 0/49 | 28.82 (1.65-504.39) | <.001 |
| EUS-FNA service | Center area≥200 m^2^ | 11/56 | 0/43 | 21.99 (1.26-384.66) | 0.002 |
| EUS-FNA service | Endoscopists ≥5 | 11/53 | 0/46 | 25.16 (1.44-440.23) | <.001 |
| EUS-FNA service | Any postgraduate physician | 11/38 | 0/61 | 51.44 (2.93-904.42) | <.001 |
| EUS-FNA service | Any senior physician | 11/75 | 0/24 | 8.74 (0.50-154.00) | 0.061 |

ORs and 95% CIs were calculated with the Haldane-Anscombe correction; *P* values are two-sided Fisher exact test results. Events are shown as events/total institutions.
